# Retrospective study of short-term complications associated with early morphine use in intubated premature infants

**DOI:** 10.1038/s41598-020-67891-w

**Published:** 2020-07-02

**Authors:** Mountasser M. Al-Mouqdad, Thanaa M. Khalil, Suzan S. Asfour

**Affiliations:** 1 Neonatal Intensive Care, NICU Department, Hospital of Paediatrics, King Saud Medical City, Al Imam Abdul Aziz Ibn Muhammad Ibn Saud, Riyadh, 12746 Saudi Arabia; 2 Obstetric and Gynecology Department, Maternity Hospital, King Saud Medical City, Riyadh, Saudi Arabia; 3 Clinical Pharmacy Department, Pharmaceutical Care Services, King Saud Medical City, Riyadh, Saudi Arabia

**Keywords:** Diseases, Medical research

## Abstract

Relieving neonatal pain is essential for the management of premature infants. Morphine is the most frequently used analgesic in neonatal intensive care. Here we report the relationship between early morphine infusion and the composite outcome of intraventricular hemorrhage and/or death in intubated premature infants. Infants (gestational age ≤ 32 weeks and birth weight < 1,500 g) intubated on admission were retrospectively evaluated in a large tertiary neonatal intensive care unit. Modified log-Poisson regression with robust variance estimator and Cox regression was applied to adjust the relative risk for infants’ outcomes. Of 420 premature infants, 230 (54.7%) received continuous morphine infusion in the first 72 h. Of these, 153 were < 28 gestational weeks; of the 190 patients who did not receive morphine, 63 were < 28 gestational weeks. The analysis revealed that infants < 28 gestational weeks who received morphine were significantly associated with an increased risk for IVH and/or death [adjusted relative risk (aRR) 1.37, 95% confidence interval (CI) 1.1–1.71)], and mortality (aRR 1.83, 95% CI 1.17–2.89). Moreover, in infants < 28 gestational weeks, survival was low in those infants who were exposed to morphine infusion in the first 72 h (hazard ratio 2.11; 95% CI 1.19–3.73). Early morphine infusion is associated with an increased risk for IVH and/or death; however, further studies are required to verify our findings.

## Introduction

Once premature infants are born and admitted in the neonatal intensive care unit (NICU), they are subjected to several invasive procedures, such as intubation, ventilation, blood extraction, and umbilical or central line insertion^1,2^. It has been proven that these procedures are painful and stressful and are associated with an increase in stress hormone levels^3^.

Neonatal pain can impact the clinical picture of patients. It alters the heart rate, blood pressure, and oxygen saturation, and consequently, it can cause intraventricular hemorrhage (IVH) in premature infants^4,5^. Increasing research has demonstrated that neonatal pain plays a vital role in the short- and long-term neurodevelopmental outcomes of premature infants^6–9^.

Therefore, theoretically, relieving pain in the early period of life will reduce the complications associated with neonatal pain and improve the potential neurodevelopmental outcome. Neonatologists have attempted to minimize the severity of pain, particularly in premature patients who are connected to mechanical ventilation, by administering sedative and analgesic medications once they are admitted. Morphine is the most commonly used analgesic medication in the NICU^10,11^.

Studies have reported that administration of morphine infusion can decrease the secretion of catecholamines and reduce the pain score^12–14^. Moreover, it can improve the synchronicity of patients’ respiration with the mechanical ventilator^15^_._ In contrast, other studies have demonstrated that morphine infusion can cause systemic hypotension^16,17^ and does not improve the respiratory outcome of intubated infants^18^. Therefore, as morphine is the most common analgesic used in the NICU, its safety and efficacy in premature infants remains disputable. This study tests the hypothesis that arbitrary initiation of morphine infusion in intubated premature infants is independently associated with an increase in the incidence of a combined outcome of IVH and/or death.

We conducted this study to investigate the relationship between initiating morphine infusion in the first 72 h after birth in intubated premature infants and the composite outcome of IVH and/or death.

## Results

**Figure 1:**
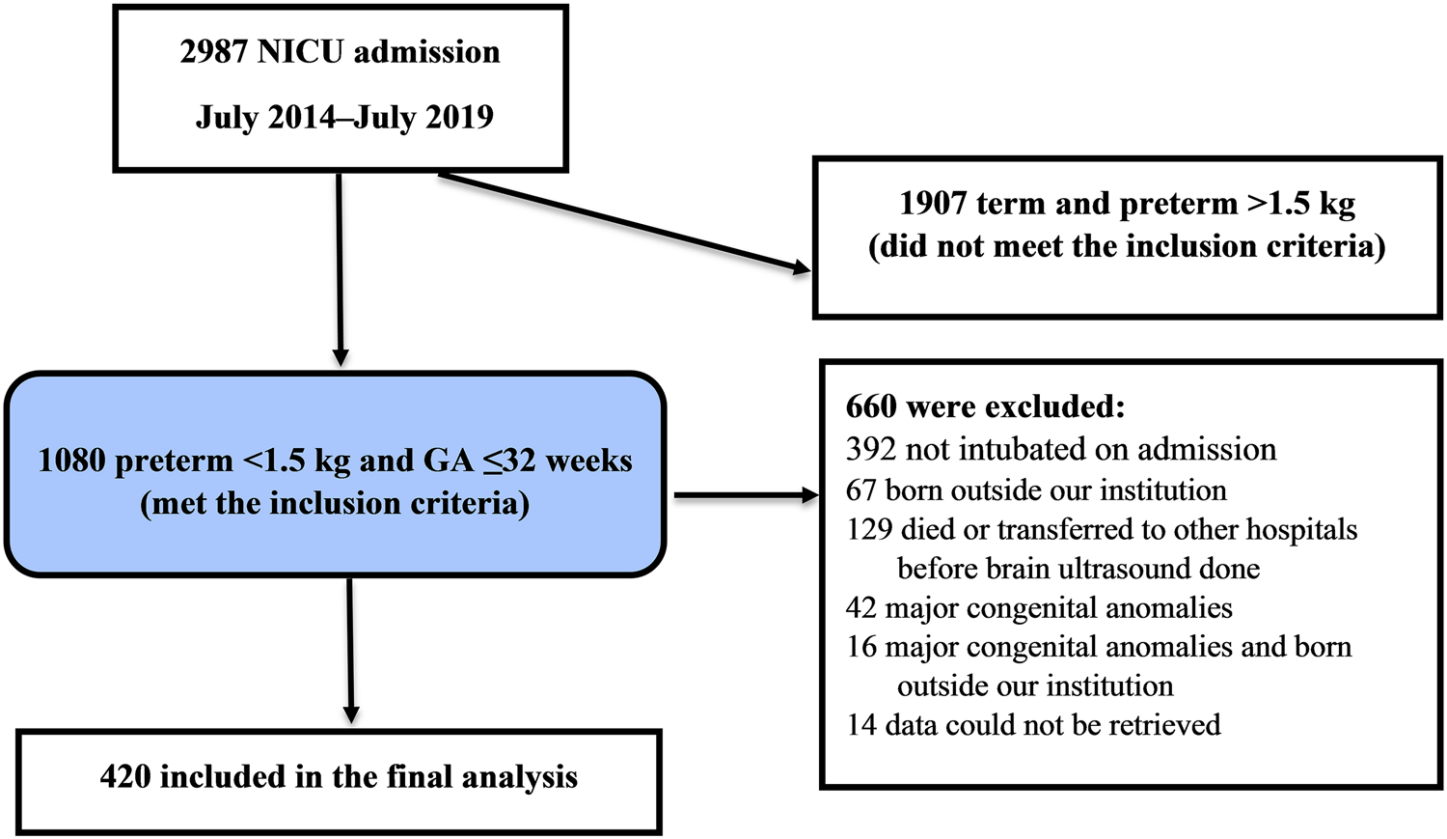
Flow chart of patient selection. *GA* gestational age, *NICU* neonatal intensive care unit.

During the study period, of 1,080 preterm infants with ≤ 32 gestational weeks and birth weight > < 1,500 g admitted to the NICU (level 3), 420 met the inclusion criteria and were eligible for inclusion in the final analysis (Fig. 1).

A total of 230 of the 420 infants (54.7%) were exposed to morphine infusion in the first 72 h after birth. Of 230 patients, 153 were aged < 28 gestational weeks. Of 190 patients who did not receive morphine, 63 were aged < 28 gestational weeks. The demographic characteristics of the mothers and infants stratified according to morphine exposure are presented in Table 1. Infants who were aged < 28 gestational weeks and received early morphine infusion had significantly lower birth weight, lower gestational age, and lower Apgar score at 1 and 5 min (P < 0.001, < 0.001, < 0.001, and 0.04, respectively).

**Table 1:** Demographic characteristics of the mothers and infants with or without exposure to morphine in the first 72 h after birth.

Gestational age	≤ 32 weeks (n = 420)			< 28 weeks (n = 216)			28–32 weeks (n = 204)		
	Exposure to morphine in the first 72 h of life			Exposure to morphine in the first 72 h of life			Exposure to morphine in the first 72 h of life		
Parameters	No (n = 190)	Yes (n = 230)	(P value)	No (n = 63)	Yes (n = 153)	(P value)	No (n = 127)	Yes (n = 77)	(P value)
Gestational age, weeks	28 (27–30)	26 (25–28)	< 0.001*	26 (26–27)	26 (24–26)	< 0.001*	29 (28–31)	29 (28–30)	0.17
Birth weight, grams	1,095 (920–1,240.25)	850 (700–1,035)	< 0.001*	910 (775–1,000)	760 (650–870)	< 0.001*	1,175 (1,055–1,315)	1,120 (1,000–1,300)	0.09
Booked	32 (16.8)	36 (15.7)	0.79	8 (12.7)	24 (15.7)	0.68	24 (18.9)	12 (15.6)	0.58
Antenatal steroid treatment	102 (53.7)	116 (50.4)	0.51	36 (57.1)	83 (54.2)	0.76	66 (52)	33 (42.9)	0.25
Cesarean section delivery	113 (59.5)	115 (50)	0.05	31 (49.2)	67 (43.8)	0.55	82 (64.6)	48 (62.3)	0.77
Maternal hypertension	39 (20.5)	37 (16.1)	0.24	12 (19)	27 (17.6)	0.85	27 (21.3)	10 (13)	0.19
Gestational diabetes mellitus	6 (3.2)	11 (4.8)	0.4	2 (3.2)	6 (3.9)	> 0.99	4 (3.1)	5 (6.5)	0.3
1-min Apgar score	5 (3–6)	4 (2–6)	< 0.001*	5 (3–6)	3 (2–5)	< 0.001*	5 (3–6)	5 (3–6)	0.21
5-min Apgar score	7 (6–8)	6 (6–7)	< 0.001*	7 (6–7)	6 (5–7)	0.04*	7 (6–8)	7 (6–8)	0.37
RDS surfactant use	152 (80)	218 (94.8)	< 0.001*	60 (95.2)	149 (97.4)	0.42	92 (72.4)	69 (89.6)	0.004*
Male sex	108 (56.8)	120 (52.2)	0.34	40 (63.5)	85 (55.6)	0.29	68 (53.5)	35 (45.5)	0.31
PDA	92 (48.4)	155 (67.4)	< 0.001*	43 (68.3)	113 (73.9)	0.41	49 (38.6)	42 (54.5)	0.03*
SNAPPE-II	25 (17–46)	34 (23–43)	0.14	40 (23, 56)	49 (37, 58)	0.24	19 (10–32)	21 (14–32)	0.69

Data are presented as median (IQR), or number (%), as appropriate.

*RDS* Respiratory distress syndrome, *PDA* patent ductus arteriosus, *SNAPPE-II* Score for Neonatal Acute Physiology with Perinatal Extension-II.

*Statistically significant at 5% level.

In the univariate analysis, we found that early initiation of morphine infusion was significantly associated with neonatal morbidity and mortality in both subgroups (Table 2). A significant association was found between infants aged < 28 gestational weeks and 28–32 gestational weeks who received morphine infusion and the composite outcome (P < 0.001 and P = 0.005, respectively). The multivariable regression analysis performed after adjusting the variables that were significant in the univariate analysis revealed that early initiation of morphine infusion remained significantly associated with the composite outcome in infants aged < 28 gestational weeks [adjusted relative risk (aRR) 1.37; 95% confidence interval (CI) 1.1–1.71)]. However, aRR revealed that there was no association between early morphine infusion and composite outcome in infants aged 28–32 gestational weeks (Table 3).

**Table 2:** Univariate analysis of neonatal morbidity and mortality stratified by gestational age and infants’ exposure to continuous morphine in the first 72 h after birth.

Gestational age	< 28 weeks (n = 216)			28–32 weeks (n = 204)		
	Exposure to morphine in the first 72 h of life			Exposure to morphine in the first 72 h of life		
Parameters	No (n = 63)	Yes (n = 153)	(P value)	No (n = 127)	Yes (n = 77)	(P value)
Composite outcome (IVH, death)	34 (54)	131 (85.6)	< 0.001*	42 (33.1)	41 (53.2)	0.005*
NEC (medical)	19 (30.2)	38 (24.8)	0.50	32 (25.2)	25 (32.5)	0.26
NEC (surgical)	6 (9.5)	26 (17)	0.21	6 (4.7)	6 (7.8)	0.37
Mortality	15 (23.8)	95 (62.1)	< 0.001*	15 (11.8)	18 (23.4)	**0.05**
BPD	42 (66.7)	77 (50.3)	0.03*	34 (26.8)	30 (39)	0.09
IVH	31 (49.2)	101 (66)	0.03*	33 (26)	32 (41.6)	0.03*
Severe IVH	61 (25.4)	63 (41.2)	0.03*	10 (7.9)	12 (15.6)	0.104
Pulmonary hemorrhage	6 (9.5)	24 (15.7)	0.28	4 (3.1)	9 (11.7)	0.03*
Inotropes	31 (49.2)	129 (84.3)	< 0.001*	36 (28.3)	39 (50.6)	0.002*
Hydrocortisone	14 (22.2)	80 (52.3)	< 0.001*	13 (10.2)	22 (28.6)	0.001*

Data are presented as number (%).

*IVH* Intraventricular hemorrhage, *NEC* necrotizing enterocolitis, *BPD* bronchopulmonary dysplasia.

*Statistically significant at 5% level.

**Table 3:** Multivariable regression for infant’s outcome stratified by gestational age and infants’ exposure to continuous morphine in the first 72 h after birth.

Outcome	< 28 weeks			28–32 weeks		
	aRR	95% CI	P value	aRR	95% CI	P value
Composite outcome (IVH, death)	1.37	1.1–1.71	0.007*	1.29	0.92–1.8	0.13
Death	1.83	1.17–2.89	0.009*	1.27	0.64–2.54	0.49
BPD	0.84	0.66–1.07	0.15	–	–	–
IVH	1.18	0.9–1.55	0.24	1.32	0.86–2.04	0.21
Severe IVH	1.5	0.97–2.34	0.07	–	–	–
Inotropes	1.45	1.13–1.88	0.004*	1.45	1.02–2.05	0.036*
Hydrocortisone	1.64	0.99–2.7	0.05	2.05	1.1–3.82	0.024*
Pulmonary hemorrhage	–	–	–	2.35	0.71–7.77	0.16

*aRR* adjusted relative risk, *CI* confidence interval, *IVH* intraventricular hemorrhage, *BPD* bronchopulmonary dysplasia.

*Statistically significant at 5% level.

We took further steps to ensure that the gestational age and birth weight do not affect the primary outcome. We performed 1:1 matching for a group of infants aged < 28 gestational weeks and who received morphine in the first 72 h of life with another group who did not received morphine. We found that early morphine infusion remained significantly associated with composite outcome and mortality (P = 0.008 and P = 0.009, respectively) (Table 4).

**Table 4:** Univariate analysis for clinical variables associated with IVH and/or death in intubated premature infants aged < 28 gestational weeks who received morphine infusion in the first 72 h of life after matching (n = 126).

Parameters	Exposure to morphine in the first 72 h of life		
	No (n = 63)	Yes (n = 63)	(P value)
GA	26 (26–7)	26 (26–26)	0.18
BW	910 (775–1,000)	850 (750–945)	0.09
Composite outcome (IVH, death)	34 (54)	49 (77.8)	0.008*
NEC (medical)	19 (30.2)	18 (28.6)	> 0.99
NEC (surgical)	6 (9.5)	12 (19)	0.2
Mortality	15 (23.8)	30 (47.6)	0.009*
PDA	43 (68.3)	47 (74.6)	0.55
IVH	31 (49.2)	39 (61.9)	0.21
Severe IVH	16 (25.4)	27 (42.9)	0.06
Pulmonary hemorrhage	6 (9.5)	13 (20.6)	0.13
Inotropes	31 (49.2)	49 (77.8)	0.002*
Hydrocortisone	14 (22.2)	23 (36.5)	0.12

*GA* gestational age, *BW* birth weight, *IVH* intraventricular hemorrhage, *NEC* necrotizing enterocolitis, *PDA* patent ductus arteriosus.

*Statistically significant at 5% level.

Table 5 shows the length of hospital stay, length of total parenteral nutrition (TPN), and invasive ventilator days among the admitted premature infants in both groups.

**Table 5:** Univariate analysis of infants’ length of hospital stay, TPN days, and invasive ventilator days stratified by gestational age and exposure to continuous morphine in the first 72 h of life.

Gestational age	< 28 weeks (n = 216)			28–32 weeks (n = 204)		
	Exposure to morphine in the first 72 h of life			Exposure to morphine in the first 72 h of life		
Parameters	No (n = 63)	Yes (n = 153)	(P value)	No (n = 127)	Yes (n = 77)	(P value)
Length of hospital stay	63 (37–84)	32 (9–81)	0.01*	42 (17–59)	44 (10–60.5)	0.86
TPN days	26 (11–52)	23 (7–43.5)	0.7	14 (7–27)	16 (7–29.5)	0.86
Invasive ventilator days	10 (2.5–24)	15 (7–28)	0.51	2.5 (1–7)	7 (4–11)	0.24

Data are presented as median (IQR).

*TPN* Total parenteral nutrition.

*Statistically significant at 5% level.

**Figure 2:**
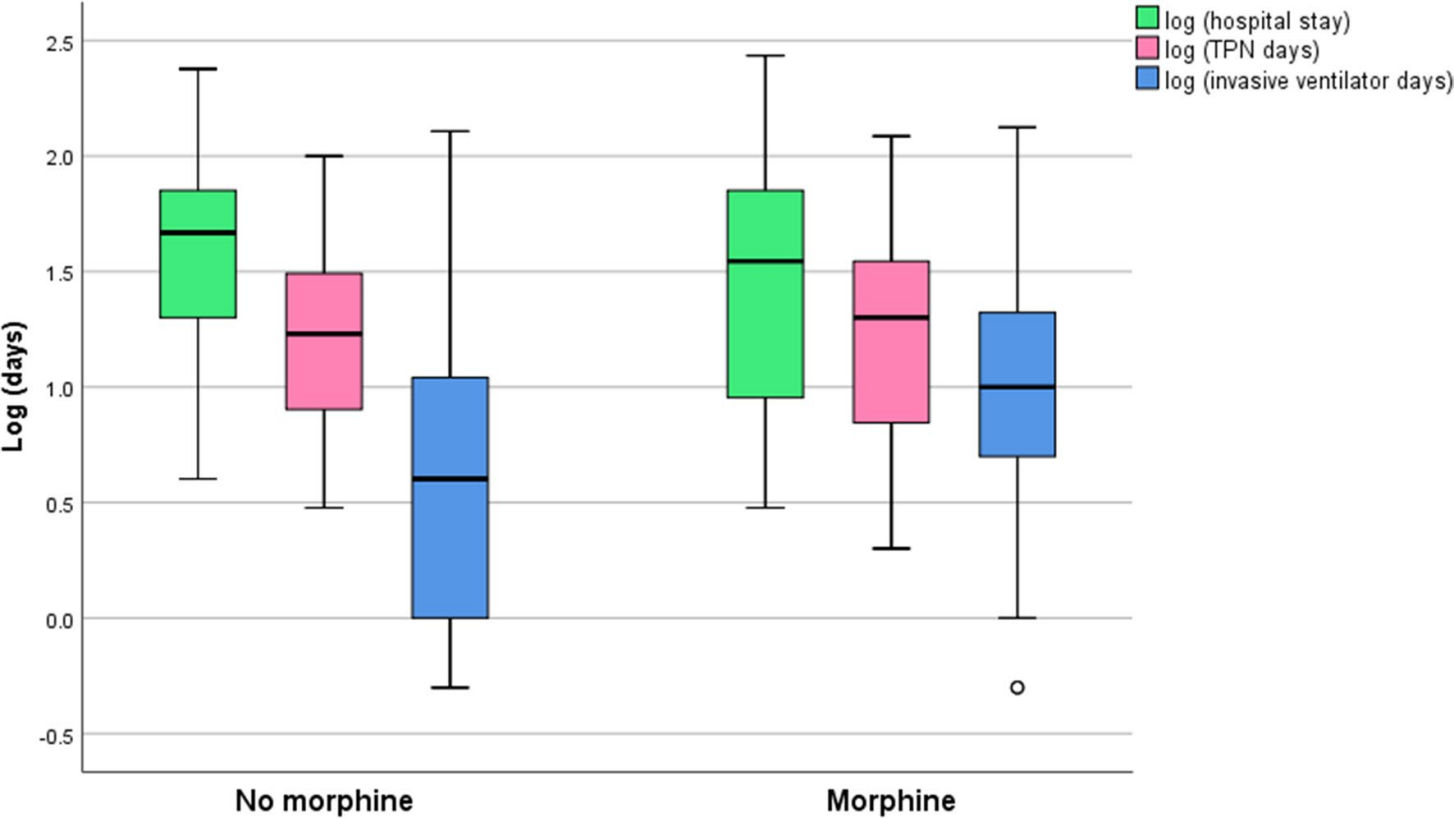
Boxplot of median hospital stay, TPN days and invasive ventilator days among infants < 28 weeks who did not receive early morphine infusion versus those who received early morphine infusion. Median values are indicated by bold horizontal lines. P = 0.01 between early morphine infusion and hospital stay. P = 0.7 and P = 0.51 respectively in both groups between early morphine infusion and TPN days and invasive ventilator days. *TPN* total parenteral nutrition.

Infant aged < 28 gestational weeks and who did not receive morphine stayed longer at hospital [median 63; interquartile range (IQR) 37–84] compared with those who received morphine infusion [median 32; IQR (9–81)] (P = 0.01) (Fig. 2).

However, after excluding the dead infants in both groups, the length of hospital stay in patients who received early morphine infusion was higher [median 85; IQR 61.75–111] compared with that in who did not receive morphine infusion [median 75; IQR (54–88.75)]. Thus, the length of hospital stay was not significantly different between the groups (P = 0.29). Moreover, there was no relationship between early exposure to morphine infusion and length of hospital stay in infants aged 28–32 gestational weeks. The median length of hospital stay in infants exposed to morphine was (median 48; IQR 23–62) and in those who were not exposed to morphine was (median 44.5; IQR 21.75–61) (P = 0.48).

In the infants aged < 28 gestational weeks, there was no significant difference in the length of invasive ventilator use and TPN among those who received morphine infusion and those who did not. Patients who received early morphine infusion stayed on invasive ventilators and TPN (median 16; IQR 7–27.25) (median 34.5; IQR 19.75–53) compared with those who did not receive morphine infusion [median 8.75; IQR (1–22.75)] (median 26; IQR 10.5–51.75) (P = 0.3 and P = 0.08, respectively). Furthermore, there was no significant difference in the length of invasive ventilator use and TPN in infants aged 28–32 gestational weeks. Patients who received early morphine infusion stayed on the invasive ventilator and TPN (median 7; IQR 3.5–10) (median 18; IQR 8–30) compared with those who did not receive morphine infusion (median 2; IQR 1–5.75) (median 13.5; IQR 7–25.75) (P = 0.17 and P = 0.56, respectively).

Multivariable negative binomial regression analysis was performed to predict the impact of several independent significant risk factors [gestational age, birth weight, 1-min and 5-min Apgar score, expressed breast milk (EBM) use, patent ductus arteriosus (PDA), and including early exposure to morphine infusion] on the length of hospital stay.

The adjusted model showed no relationship between morphine exposure and length of hospital stay among admitted infants < 28 gestational weeks (aRR 0.83, 95% CI 0.63–1.08, P = 0.16).

**Figure 3:**
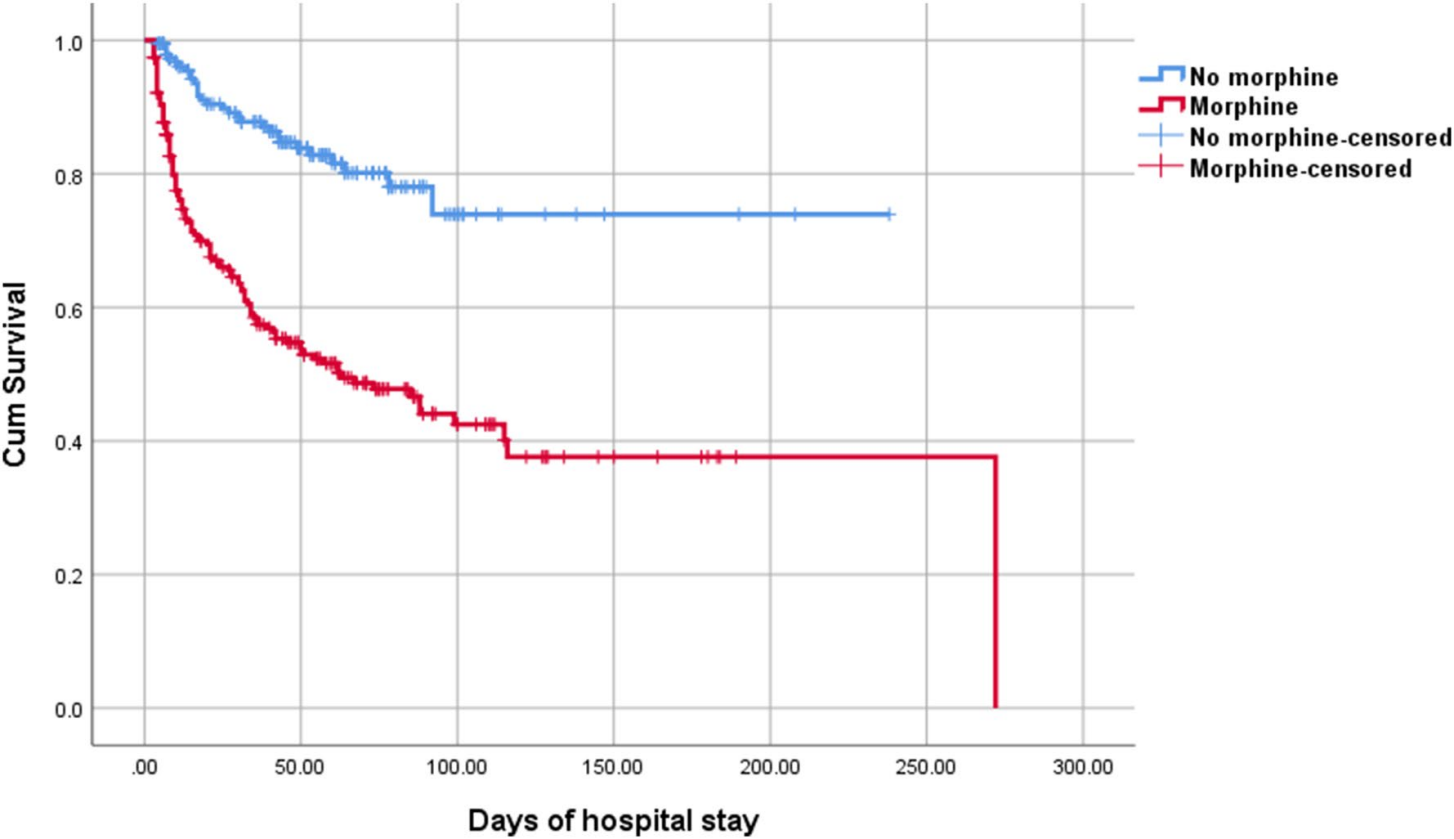
Kaplan–Meier curves showing the relationship between early exposure to morphine and survival among all infants, log-rank test, P < 0.001.

Figure 3 shows the Kaplan–Meier curves of the relationship between early exposure to morphine and survival among all infants (log-rank test, P < 0.001). Multivariable Cox regression curve adjusted for significant potential confounders (gestational age, birth weight, 1-min and 5-min Apgar scores, IVH, severe IVH, EBM use, PDA, and PET) showed a significant association between infants who received morphine infusion in the first 72 h after birth and survival, and the hazard ratios (HRs) for death increased by 1.97-fold (95% CI 1.27–3.08) compared with that of infants who did not receive early morphine infusion.

**Figure 4:**
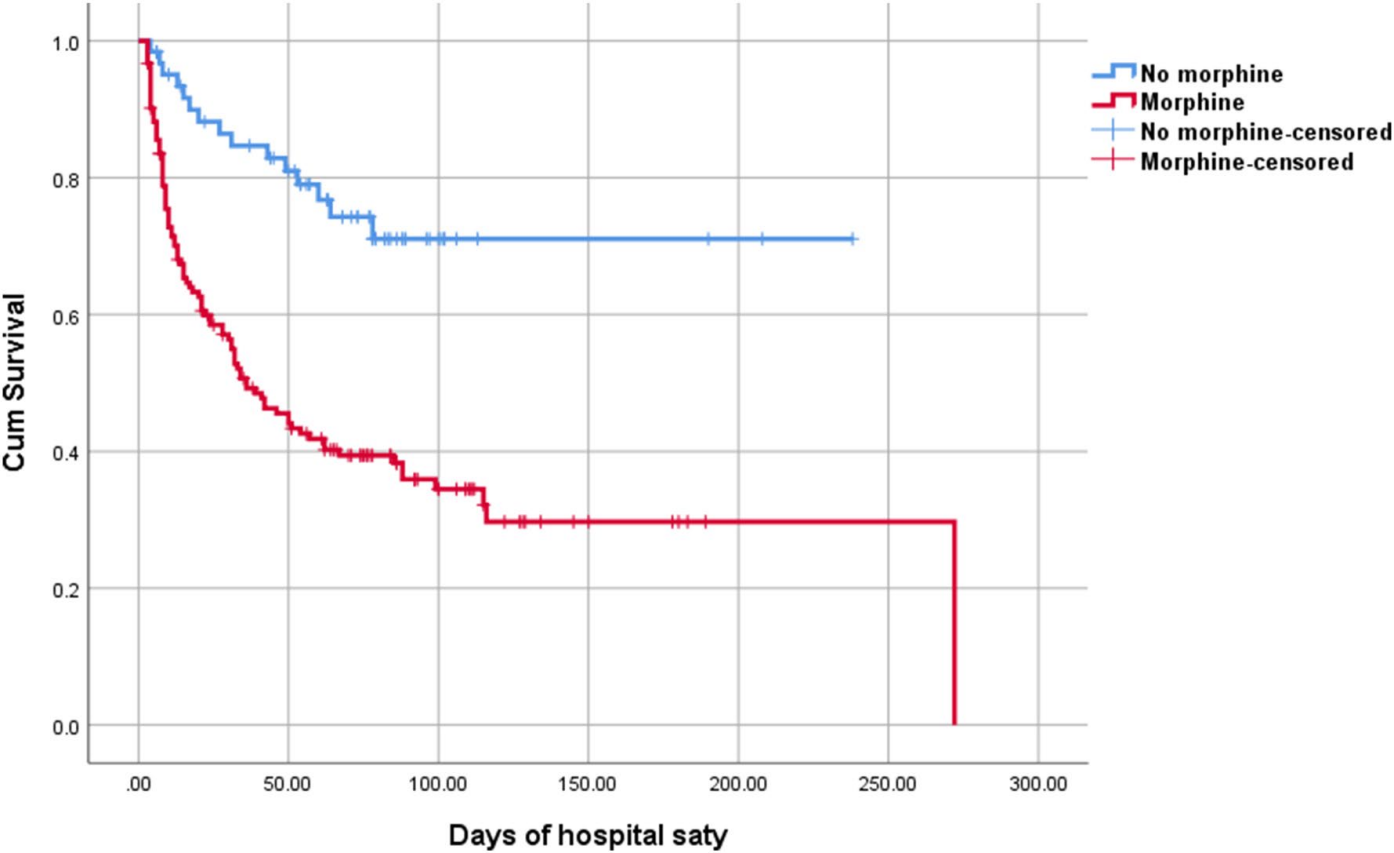
Kaplan–Meier curves showing the relationship between early exposure to morphine and survival among infants aged < 28 gestational weeks, log-rank test, P < 0.001.

Figure 4 shows Kaplan–Meier curves of the relationship between early exposure to morphine and survival among infants aged < 28 gestational weeks (log-rank test, P < 0.001). In infants aged < 28 gestational weeks, the multivariable Cox regression curve adjusted for significant potential confounders (gestational age, birth weight, 1-min and 5-min Apgar scores, IVH, severe IVH, and EBM use) showed that in infants who received morphine infusion in the first 72 h after birth, the HRs for death increased by 2.11-fold (95% CI 1.19–3.73) compared with that of infants who did not receive early morphine infusion.

**Figure 5:**
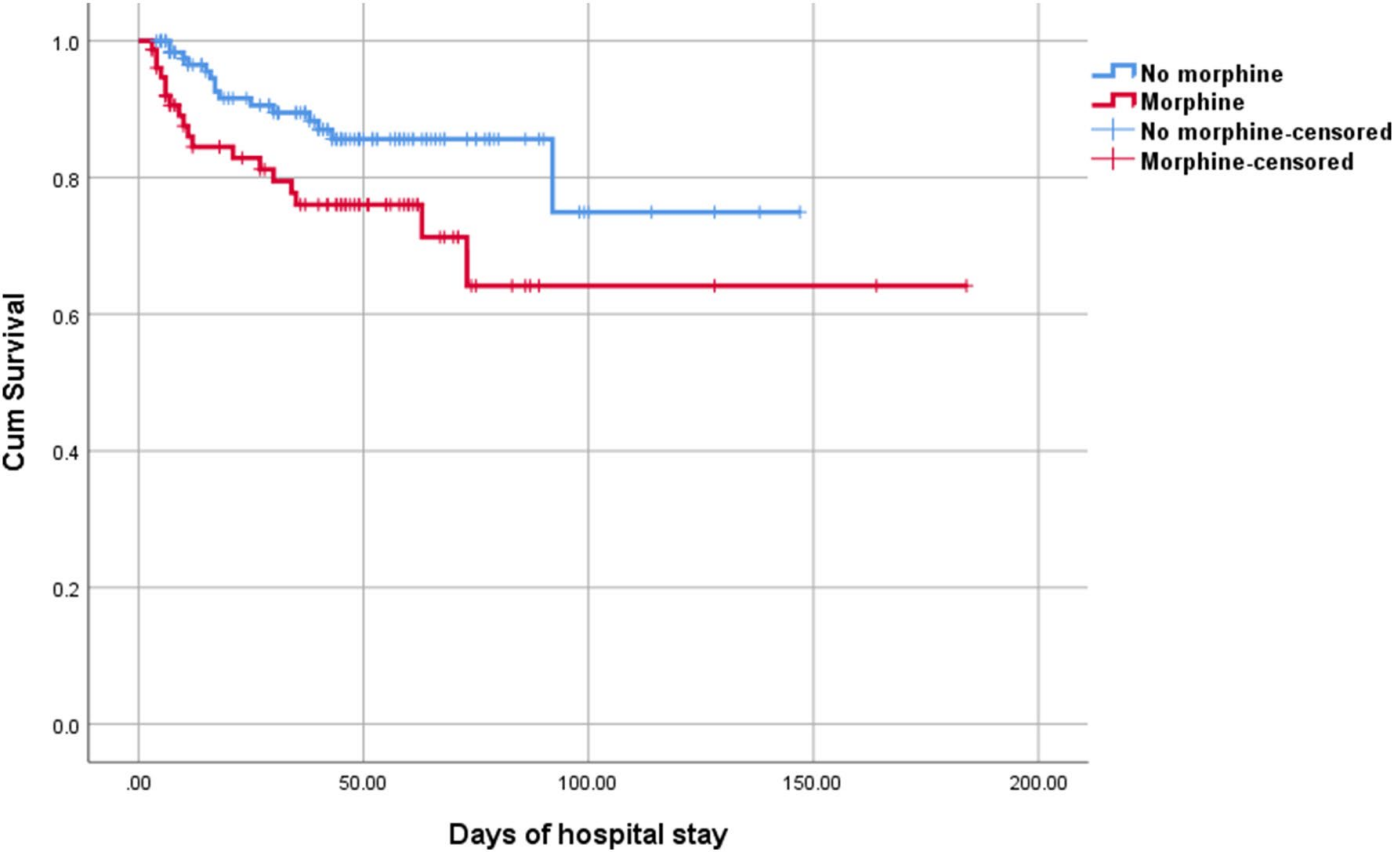
Kaplan–Meier curves showing the relationship between early exposure to morphine and survival among infants aged 28–32 gestational weeks, log-rank test, P = 0.024.

In addition, Kaplan–Meier curves showed the relationship between early exposure to morphine and survival among infants aged 28–32 gestational weeks (log-rank test, P = 0.02) (Fig. 5). However, the multivariable Cox regression adjusted for potential confounders (1-min Apgar scores and PDA) in infants aged 28–32 gestational weeks showed that there was no association between early morphine infusion in the first 72 h after birth and death (HR 1.92, 95% CI 0.96–3.82).

## Discussion

In the present study, we found that initiation of continuous morphine infusion therapy in the first 72 h after birth in intubated premature infants aged < 28 gestational weeks increased the risk for IVH and/or death after controlling for confounder variables. Moreover, it increased the possibility of developing systemic hypotension in all premature infants who are intubated and aged ≤ 32 gestational weeks, which is a well-known side effect of morphine administration^17,19^ and consequently increased the use of inotropes and hydrocortisone for treating systemic hypotension. We assumed that early initiation of continuous morphine infusion in intubated premature infants compromises the systemic blood pressure, prompting the neonatologist to establish inotropes, which in turn increases the fluctuation of cerebral blood flow and causes brain injury.

A large randomized clinical trial investigated whether morphine decreases the incidence of brain injury and/or death^17^. The “NEOPAIN” study reported the following two findings: first, early continuous morphine infusion was associated with an increased risk for brain injury and/or death (P = 0.03) and severe IVH (P = 0.02); second, intermittent morphine boluses dramatically increased the rate of death and brain injury (P = 0.0001). Our study results of the intubated premature infants aged < 28 gestational weeks are consistent with the “NEOPAIN” trial results; therefore, continuous morphine infusion in the first 72 h after birth may increase the risk of developing brain injury and/or death.

Moreover, some studies found that assisted mechanical ventilation aggravates the pain score in premature infants^3,20^. Therefore, a good number of neonatologists routinely initiate morphine infusion early in premature infants who require assisted ventilation. Studies have found that morphine improves the synchronization between the intubated infants and the mechanical ventilation and, as a consequence, reduces the number of days of ventilation^15,21^. In these studies, the new ventilation strategies were not available, and they had a small sample size and included infants who were aged > 32 gestational weeks.

In contrast, the “NEOPAIN” trial found that morphine prolonged the duration of mechanical support in extremely premature infants^22^.

Our study finding is not consistent with the latter one, i.e., there was no change in the ventilator duration in the intubated premature infants’ group who received continuous morphine infusion in the first 72 h after birth. Therefore, the initiation of morphine infusion on admission has no particular pulmonary benefit, which was also the conclusion of a meta-analysis Bellu et al.^23^.

Another aspect in our study is that early infusion of morphine was not associated with an increased incidence of gastrointestinal pathology such as NEC and the period of TPN among the survivors. In this regard, our results are compatible with those of the “NEOPAIN” trial, in which morphine exposure did not affect NEC, either medical or surgical NEC; however, it prolonged the duration to reach the full feed status^24^.

Apparently, administering systemic morphine infusion to intubated premature infants in the early period of life increases the risk of mortality twofold compared with that in those who do not receive continuous morphine infusion. This observed relationship between early infusion of morphine and mortality risk corresponds with the results of studies stating that morphine enhances the apoptotic death of cells in the immune and nervous systems, which would negatively influence the developmental outcome of premature infants^25–28^.

In our opinion, the drawbacks of administering continuous morphine infusion outweigh the advantages; therefore, initiating early morphine infusion should be restricted and applied according to very clear guidelines. Furthermore, nonpharmacological interventions such as skin-to-skin care, music therapy, and parental attendance can be implemented. A recent systemic review has shown that kangaroo mother care can make the patient more comfortable by reducing the pain score and the episodes of crying^28^. Similarly, music therapy can be beneficial in minimizing the stress and pain caused during the procedures^29^.

There are some limitations to our study. First, because this study was retrospective, it was difficult to control for all clinical variables using statistical methods. However, those variables that were related to disease severity were adjusted. Furthermore, by applying the Cox regression model, early initiation of morphine infusion was found to be associated with increased risk of mortality and developing IVH in premature infants aged < 28 gestational weeks. Second, the attending neonatologists who ordered continuous morphine infusion did not look at the pain score and did not use it as a score to finalize or control the dose of morphine infusion. Third, we did not focus on the dose of morphine infusion as it was variable and ranged between 10 and 20 µg/kg/h without using morphine boluses; however, some studies have reported the lack of relationship between the clinical efficacy and the concentration of morphine infusion^31–32^. We do not use other analgesics or sedative medications such as fentanyl or midazolam on admission, and therefore, a discussion about their efficacy in terms of outcomes is not in the scope of this study.

Our study has several strong aspects. First, we included only those premature inborn infants who required intubation within the first 72 h of life. Second, head ultrasound findings were reviewed by a paediatric radiologist who was not aware of the aim of the study. Third, because we had a good number of patients in each group, we attempted to stratify the outcomes using the gestational age, making the results more specific. Fourth, our study findings can serve as a reminder to NICU practitioners regarding the serious complications of early use of systemic analgesia as there are limited data regarding this aspect. In conclusion, establishing early morphine infusion in intubated premature infants is associated with an increased incidence of the composite outcome of IVH and/or death in infants who are aged < 28 gestational weeks. Large, well-designed, randomized controlled trials are required to address the sequelae of early initiation of sedation and analgesia in intubated premature infants.

## Methods

### Study design

This study was a retrospective cohort review of the charts of premature infants (gestational age ≤ 32 weeks; birth weight < 1,500 g) who were intubated and admitted to the NICU of King Saud Medical City (KSMC) tertiary referral center from July 2014 to July 2019.

The NICU has an average annual admission of 1,100 patients, including NICU levels 2 and 3. This study was conducted in accordance with the Declaration of Helsinki and Good Pharmacoepidemiology Practices guidelines and was approved by the medical ethical review committee of KSMC with a waiver of consent (reference number H1RI-25-Feb19-01).

### Definitions

#### Intraventricular hemorrhage

IVH was classified into grades I–IV according to the IVH classification described by Papile et al. IVH was diagnosed based on the findings of head ultrasound performed between days 5 and 7 of life after birth^33,34^. All ultrasound findings were reviewed by an expert pediatric radiologist. Transducers of 7.5 and 10 MHz (LOGIQ e; GE Medical Systems Co., Ltd., Jiangsu, China) were used to perform ultrasound in the sagittal and coronal planes.

Bronchopulmonary dysplasia (BPD) was defined as the need for respiratory support at 36 weeks of corrected gestational age^35^.

#### Necrotizing enterocolitis (NEC)

This includes all Bell Staging Criteria^36^.

### Inclusion and exclusion criteria

Infants included in this study were born at KSMC at ≤ 32 gestational weeks with a birth weight of < 1,500 g, admitted to the NICU, and intubated and received continuous infusion of morphine within the first 72 h of life. In the control group, intubated patients did not receive morphine within the first 72 h of life during the same study period. Gestational age was categorized into two groups: < 28 gestational weeks and 28–32 gestational weeks.

We excluded all non-intubated patients on admission, those with major congenital anomalies, those who were not born at KSMC, those who had congenital infection, those who developed early onset of sepsis, or those for whom data could not be retrieved.

A sample size of 420 premature infants was analysed to detect differences in the short-term outcome.

### Data collection and follow-up

Patients’ charts from NICU admission until discharge or death were reviewed. Demographic and clinical data and outcome data of all infants were obtained. Maternal data, including the presence of gestational diabetes mellitus, maternal hypertension, antenatal steroid treatment, and mode of delivery, were also obtained.

### Study outcomes

The primary outcome of this study was the composite outcome, comprising IVH and/or mortality. The secondary outcomes were any grade of IVH, severe IVH (grade III or IV), NEC (medical or surgical), BPD, and infant mortality.

### Statistical analysis

Before starting the analysis, the dataset was checked for missing variables. Data were analyzed using a statistical software package (Statistical Package for the Social Sciences, version 25.0, SPSS Inc., Chicago, IL, USA).

Descriptive statistics, including median, (IQR), frequency, and percentages, were used to describe the maternal and neonatal variables.

The Fisher’s exact test was used to determine the association between categorical variables. The Mann–Whitney U test was used for ordinal qualitative variables [gestational age, birth weight, Apgar score, and the Score for Neonatal Acute Physiology with Perinatal Extension-II (SNAPP-II)]. For continuous variables, the unpaired Student’s *t*-test was used; when data were not normally distributed, the Mann–Whitney U test was performed. The Kolmogorov–Smirnov test and a visual inspection of histograms were performed to verify the normality of distribution of the quantitative variables.

To analyse the association between early exposure to morphine infusion and outcomes, a univariate relative risk analysis was first conducted on the recorded variables [gestational age, birth weight, gender, 1-min Apgar, 5-min Apgar, SNAPPE-II, surfactant use, maternal hypertension, antenatal steroid treatment, mode of delivery, (PDA), and (EBM)] because we considered those to be potential confounders^38–39^. All factors with a P value of < 0.05 in the univariate analysis were considered for inclusion in the final multivariable regression model. Modified log-Poisson regression in generalized linear models with robust variance estimator (Huber-White) was applied for univariate relative risk analysis and to the models to adjust the relative risk for infants’ morbidity and mortality outcomes.

A negative binomial regression analysis was conducted to compare the effect of early morphine infusion exposure on dependent variables [days on ventilator, days of (TPN), and length of hospital stay].

Kaplan–Meier survival curve with log rank test was used to compare survival among those who received morphine versus those who did not receive morphine. Multivariable Cox regression analysis was used to analyse the HRs for death with 95% CIs. Independent variables for the models were selected from variables significantly related to death in a univariate analysis. All statistical tests were 2-tailed, and P values of < 0.05 were considered to be statistically significant.

## Data Availability

The datasets analysed during this study are available from the corresponding author on reasonable request.

## References

[1] Carbajal R (2008). Epidemiology and treatment of painful procedures in neonates in intensive care units. JAMA.

[2] Cruz MD, Fernandes AM, Oliveira CR (2016). Epidemiology of painful procedures performed in neonates: A systematic review of observational studies. Eur. J. Pain.

[3] Barker DP, Rutter N (1996). Stress, severity of illness, and outcome in ventilated preterm infants. Arch. Dis. Child. Fetal Neonatal Ed..

[4] Anand KJ (1998). Clinical importance of pain and stress in preterm neonates. Biol. Neonate.

[5] Pineles BL, Sandman CA, Waffarn F, Uy C, Davis EP (2007). Sensitization of cardiac responses to pain in preterm infants. Neonatology.

[6] Bouza H (2009). The impact of pain in the immature brain. J. Matern. Fetal Neonatal Med..

[7] Walker SM (2017). Translational studies identify long-term impact of prior neonatal pain experience. Pain.

[8] Smith GC (2011). Neonatal intensive care unit stress is associated with brain development in preterm infants. Ann. Neurol..

[9] Steinhorn R (2015). Neonatal morphine exposure in very preterm infants-cerebral development and outcomes. J. Pediatr..

[10] Clark RH, Bloom BT, Spitzer AR, Gerstmann DR (2006). Reported medication use in the neonatal intensive care unit: Data from a large national data set. Pediatrics.

[11] Kumar P (2008). Medication use in the neonatal intensive care unit: Current patterns and off-label use of parenteral medications. J. Pediatr..

[12] Quinn MW (1992). Effect of morphine and pancuronium on the stress response in ventilated preterm infants. Early Hum. Dev..

[13] Quinn MW (1993). Randomised double-blind controlled trial of effect of morphine on catecholamine concentrations in ventilated pre-term babies. Lancet.

[14] Simons SH (2005). Randomised controlled trial evaluating effects of morphine on plasma adrenaline/noradrenaline concentrations in newborns. Arch. Dis. Child Fetal Neonatal Ed..

[15] Dyke MP, Kohan R, Evans S (1995). Morphine increases synchronous ventilation in preterm infants. J. Paediatr. Child Health.

[16] Carbajal R (2005). Morphine does not provide adequate analgesia for acute procedural pain among preterm neonates. Pediatrics.

[17] Anand KJ (2004). Effects of morphine analgesia in ventilated preterm neonates: Primary outcomes from the NEOPAIN randomised trial. Lancet.

[18] Simons SH (2003). Routine morphine infusion in preterm newborns who received ventilatory support: A randomized controlled trial. JAMA.

[19] Hall RW (2005). Morphine, hypotension, and adverse outcomes among preterm neonates: Who's to blame? Secondary results from the NEOPAIN trial. Pediatrics.

[20] Hall RW, Boyle E, Young T (2007). Do ventilated neonates require pain management?. Semin. Perinatol..

[21] Jiang HH (2012). Clinical evaluation of the effects of morphine in mechanical ventilation of neonates. Zhonghua Er Ke Za Zhi.

[22] Bhandari V (2005). Morphine administration and short-term pulmonary outcomes among ventilated preterm infants. Pediatrics.

[23] Bellu R, de Waal K, Zanini R (2010). Opioids for neonates receiving mechanical ventilation: A systematic review and meta-analysis. Arch. Dis. Child. Fetal Neonatal Ed..

[24] Menon G (2008). Morphine analgesia and gastrointestinal morbidity in preterm infants: Secondary results from the NEOPAIN trial. Arch. Dis. Child. Fetal Neonatal Ed..

[25] Singhal PC (2002). Role of p38 mitogen-activated protein kinase phosphorylation and Fas-Fas ligand interaction in morphine-induced macrophage apoptosis. J. Immunol..

[26] Goswami R, Dawson SA, Dawson G (1998). Cyclic AMP protects against staurosporine and wortmannin-induced apoptosis and opioid-enhanced apoptosis in both embryonic and immortalized (F-11kappa7) neurons. J. Neurochem..

[27] Kocek M, Wilcox R, Crank C, Patra K (2016). Evaluation of the relationship between opioid exposure in extremely low birth weight infants in the neonatal intensive care unit and neurodevelopmental outcome at 2 years. Early Hum. Dev..

[28] Johnston C (2017). Skin-to-skin care for procedural pain in neonates. Cochrane Database Syst. Rev..

[29] Hartling L (2009). Music for medical indications in the neonatal period: A systematic review of randomised controlled trials. Arch. Dis. Child. Fetal Neonatal Ed..

[30] Scott CS (1999). Morphine pharmacokinetics and pain assessment in premature newborns. J. Pediatr..

[31] Saarenmaa E, Neuvonen PJ, Rosenberg P, Fellman V (2000). Morphine clearance and effects in newborn infants in relation to gestational age. Clin. Pharmacol. Ther..

[32] Anand KJ (2008). Morphine pharmacokinetics and pharmacodynamics in preterm and term neonates: Secondary results from the NEOPAIN trial. Br. J. Anaesth..

[33] Ballabh P (2010). Intraventricular hemorrhage in premature infants: Mechanism of disease. Pediatr. Res..

[34] Huvanandana J (2017). Prediction of intraventricular haemorrhage in preterm infants using time series analysis of blood pressure and respiratory signals. Sci. Rep..

[35] Jobe AH, Bancalari E (2001). Bronchopulmonary dysplasia. Am. J. Respir. Crit. Care Med..

[36] Bell MJ (1978). Neonatal necrotizing enterocolitis. Therapeutic decisions based upon clinical staging. Ann. Surg..

[37] Hintz SR (2005). Neurodevelopmental and growth outcomes of extremely low birth weight infants after necrotizing enterocolitis. Pediatrics.

[38] Kurlat I, Stoll BJ, McGowan JE (1989). Time to positivity for detection of bacteremia in neonates. J. Clin. Microbiol..

[39] Kristof K, Kocsis E, Nagy K (2009). Clinical microbiology of early-onset and late-onset neonatal sepsis, particularly among preterm babies. Acta Microbiol. Immunol. Hung..

